# Single-Cell RNA-seq Reveals Characteristics of Malignant Cells and Immune Microenvironment in Subcutaneous Panniculitis-Like T-Cell Lymphoma

**DOI:** 10.3389/fonc.2021.611580

**Published:** 2021-03-18

**Authors:** Zifeng Li, Hongsheng Wang, Rui Dong, Jie Man, Li Sun, Xiaowen Qian, Xiaohua Zhu, Ping Cao, Yi Yu, Jun Le, Yang Fu, Ping Wang, Wenjin Jiang, Chen Shen, Yangyang Ma, Lian Chen, Yaochen Xu, Jiantao Shi, Hui Zhang, Maoxiang Qian, Xiaowen Zhai

**Affiliations:** ^1^ Department of Hematology and Oncology, Children’s Hospital of Fudan University, National Children's Medical Center, Shanghai, China; ^2^ Department of Pediatric Surgery, Children’s Hospital of Fudan University, National Children's Medical Center, Shanghai, China; ^3^ Department of Rheumatism and Immunology, Children’s Hospital of Fudan University, National Children's Medical Center, Shanghai, China; ^4^ Department of Pathology, Children’s Hospital of Fudan University, National Children's Medical Center, Shanghai, China; ^5^ Shanghai Institute of Biochemistry and Cell Biology, Center for Excellence in Molecular Cell Science, Chinese Academy of Sciences, Shanghai, China; ^6^ Department of Hematology/Oncology, Guangzhou Women and Children’s Medical Center, Guangzhou, China; ^7^ Institute of Pediatrics, Children’s Hospital of Fudan University, National Children's Medical Center, and the Shanghai Key Laboratory of Medical Epigenetics, Institutes of Biomedical Sciences, Fudan University, Shanghai, China

**Keywords:** single-cell RNA-seq (scRNA-seq), T cell malignancies, pediatric oncology, molecular diagnoses, subcutaneous panniculitis- like T-cell lymphoma

## Abstract

**Background:**

Subcutaneous panniculitis-like T-cell lymphoma (SPTCL) is a malignant primary T-cell lymphoma that is challenging to distinguish from autoimmune disorders and reactive panniculitides. Delay in diagnosis and a high misdiagnosis rate affect the prognosis and survival of patients. The difficulty of diagnosis is mainly due to an incomplete understanding of disease pathogenesis.

**Methods:**

We performed single-cell RNA sequencing of matched subcutaneous lesion tissue, peripheral blood, and bone marrow from a patient with SPTCL, as well as peripheral blood, bone marrow, lymph node, and lung tissue samples from healthy donors as normal controls. We conducted cell clustering, gene expression program identification, gene differential expression analysis, and cell-cell interaction analysis to investigate the ecosystem of SPTCL.

**Results:**

Based on gene expression profiles in a single-cell resolution, we identified and characterized the malignant cells and immune subsets from a patient with SPTCL. Our analysis showed that SPTCL malignant cells expressed a distinct gene signature, including chemokines families, cytotoxic proteins, T cell immune checkpoint molecules, and the immunoglobulin family. By comparing with normal T cells, we identified potential novel markers for SPTCL (e.g., *CYTOR, CXCL13, VCAM1, and TIMD4*) specifically differentially expressed in the malignant cells. We also found that macrophages and fibroblasts dominated the cell-cell communication landscape with the SPTCL malignant cells.

**Conclusions:**

This work offers insight into the heterogeneity of subcutaneous panniculitis-like T-cell lymphoma, providing a better understanding of the transcription characteristics and immune microenvironment of this rare tumor.

## Introduction

Subcutaneous panniculitis-like T-cell lymphoma (SPTCL) is a rare primary cutaneous lymphoma of mature cytotoxic T cells arising primarily in the skin without the evidence of extracutaneous involvement. According to the 2016 World Health Organization (WHO) and 2018 World Health Organization-European Organization for Research and Treatment of Cancer (WHO-EORTC) classification, SPTCL is defined as subcutaneous lymphomas with an α/β T cell phenotype and neoplastic T cells expressing CD3, CD8, and cytotoxic proteins (GZMB, TIA-1, perforin) (1, 2). Both children (3) and adults can be affected, with a median age at diagnosis of 36 years and female gender bias (4). In a cohort of pediatric patients (3), the median age at diagnosis was 8 years (5 months to 21 years) with a male to female ratio of 1:1.7. The disease response to therapy is usually favorable, with a 5-year survival of more than 80% (5).

However, the clinical manifestations and pathological features of SPTCL are similar to those of benign panniculitis, lupus erythematosus profundus (LEP), and various autoimmune disorders, thus SPTCL is frequently misdiagnosed at the early stage (6). The long diagnosis period and high misdiagnosis rate may affect the prognosis and survival of patients. Although recent studies have provided insight into pathways that may be important to the pathogenesis of this disease (5, 7–11), additional investigations are required to better understand the profile and ecosystem of SPTCL.

Here, we conducted single-cell RNA sequencing (scRNA-seq) to decipher SPTCL at an unprecedented transcriptomic resolution for matched subcutaneous lesion tissue, peripheral blood, and bone marrow from a patient with SPTCL, as well as peripheral blood, bone marrow, lymph node, and lung tissue samples from healthy donors as normal controls. Using this dataset, we investigated the ecosystem of SPTCL and identified novel markers of SPTCL that may advance the detection and diagnosis of this disease.

## Methods

### Patient

A male patient diagnosed with SPTCL was recruited from the Children’s Hospital of Fudan University in the Department of Hematology and Oncology. At the time of sample collection, the patient was 22 months old with SPTCL.

This study was approved by the Medical Ethics Committee of the Children’s Hospital of Fudan University institutional review board and conducted under the Declaration of Helsinki principles (approval reference: No (2020). 307). Informed written consent was obtained from the parents before inclusion in the study.

### Healthy Donors

Healthy donors’ datasets were downloaded from the Gene Expression Omnibus (GEO, accession number: GSE126030) (12). The samples were obtained from deceased, brain-dead donors at the time of organ acquisition for clinical transplantation. Donors were free of chronic disease, cancer, and chronic infections such as Hepatitis B, C, and HIV. The mononuclear cells were isolated from human lungs (LG), lymph nodes (LN), bone marrow (BM), and blood, and the untouched CD3+ T cells were enriched from single-cell suspensions of all tissues and blood using magnetic negative selection (MojoSort Human CD3+ T cell Isolation Kit; BioLegend) (12).

### Single-Cell RNA Sequencing

Experimental procedures followed established techniques using the Chromium Single Cell 3’ Library V3 kit (10x Genomics). Briefly, mononuclear cells from enzymatically digested subcutaneous lesion biopsies and bone marrow, as well as peripheral blood by density gradient centrifugation using Lymphocyte Separation Medium, were loaded into the Chromium instrument (10X Genomics), and the resulting barcoded cDNAs were used to construct libraries. RNA-seq was performed on each sample (approximately 200 million reads/sample). Raw sequence data were converted into FASTQs using the Illumina bcl2fastq software. FASTQ files were aligned to the human genome (GRCh38) using the *CellRanger* v3.0.1 (10x Genomics) pipeline according to the manufacturer’s instructions.

### Single-Cell Data Processing and Analysis

Initial data processing of scRNA-seq for peripheral blood (n = 6,463), bone marrow (n = 11,027), and subcutaneous lesion tissue (n = 19,247) from the patient were performed using Python 3.6 and the Single Cell Analysis in Python (*Scanpy*) (v1.4.6) (13) unless otherwise stated. Healthy donors’ scRNA-seq data were also processed in the same way. Individual cells were filtered based on the total number of genes expressed and the percentage of mitochondrial reads. The cells were included with genes greater than 200 but less than 6,000, and the percentage of mitochondrial reads less than 10%. Genes detected in fewer than three cells were filtered out. Read counts of qualified cells were normalized using the deconvolution method implemented in the R package *Scran* (v3.11) (14) and in-transformed.

### Single-Sample Analysis

For visualization, a UMAP was calculated by computing the single-cell neighborhood graph (kNN-graph) on the specific principal components using 15 neighbors. The number of principal components utilized in the neighborhood graph was based on the standard deviations of the top 30 principal components. The Leiden graph-clustering method was used to cluster the neighborhood graph of cells.

Cell types were manually assigned to the clusters from the Leiden graph-clustering by comparing the mean expression of known markers across cells in a cluster. Markers used to type cells included *CD19, MS4A1, CD79A* (B cells), *CD2, CD3, CD4, CD8* (T cells), *CCR7, IL7R, LEF1, SELL* (naive T cells), *CD44, CXCR3* (memory T cells), *IL2RA, FOXP3, IKZF2* (Tregs), *CXCR5, BCL6, KLRB1, CCR4, TBX21, GATA3* (Th cells), *NCAM1, NKG7* (NK cells), *CD14, FCGR3A, ITGAM, CD68, ITGB2, ADGRE1, LYZ* (macrophages), *IRF8, CLEC4C* (dendritic cells), *DPP4, TAGLN, COL1A1, PDGFRA* (fibroblasts), and *CD34* (progenitor).

A consensus non-negative matrix factorization (cNMF) algorithm (15) was employed to identified gene expression programs (GEPs) following the protocol on Github https://github.com/dylkot/cNMF. The GEPs obtained were subjected to Gene Ontology (GO) and KEGG analysis using the R package *clusterProfiler* (v3.11) (16).

### Integration Sample Analysis

We combined the data generated from isolated cells with CD3 and CD8 positive from peripheral blood (n= 1,812), bone marrow (n=1,143), and subcutaneous lesion tissue (n=5,956) of the patient, and healthy donors (n=13,494) to conduct integration analysis. The Scanorama algorithm (17) was applied to correct the combined dataset for technical batch effects. All reduced dimensions were the same as that in the single-sample analysis. Partition-based graph abstraction (PAGA) was calculated by *Scanpy*.

The top 100 correlated genes were defined as a GEP, and their average relative expression was calculated as a GEP cell score (18). The reference set was randomly sampled from the gene pool for each binned expression value. The number of reference genes to be sampled from each bin was 100.

The Wilcoxon rank-sum test was used to estimate and identify differentially expressed genes. The novel markers utilized a default threshold of 2 for average fold change and a filter for the minimum delta percent of cells ([X (percentage of cluster1) – X (percentage of cluster2)]/X (percentage of cluster1) * 100) greater than 90%.

### InferCNV Analysis

Raw gene expression data were extracted from the Scanpy object as recommended in the “Using 10x data” section (inferCNV of the Trinity CTAT Project, https://github.com/broadinstitute/inferCNV). Normal reference cells were identified from annotated Leiden clusters as naïve T cells. Tumor cells were identified as malignant-like cells in Leiden clusters. The inferCNV analysis was performed following the tutorial (https://github.com/broadinstitute/inferCNV/wiki) with parameters including default settings.

### Cell-Cell Ligand-Receptor Interactions

Cell-cell ligand-receptor interactions were inferred using the *CellPhoneDB* (v2.0.0) method in Python (19). The lower cutoff for the expression proportion of any ligand or receptor in a given cell type was set to 10%, and the number of permutations was set to 1000.

### Whole-Exome Sequencing and Analysis

DNA was extracted from paraffin-embedded (FFPE) SPTCL tissue for whole-exome sequencing (WES). The Agilent SureSelect Human All Exon V6 kit was used for exome capture and library preparation. Paired-end sequencing (2 x 150 bp read length) was performed using the Illumina NovaSeq platform. Reads were mapped to the human genome (GRCh37) reference sequence by the Burrows-Wheeler aligner (bwa mem) algorithm (version 0.7.17) (20). The data processing, including indel realignment, marking duplicates, and recalibrating base quality scores, were performed according to the GATK best practices using GATK (version 3.7) (21) and Picard tools (version 2.18.25, http://broadinstitute.github.io/picard). Variants in the *HAVCR2* gene were manually checked using the Integrative Genomics Viewer (IGV) with the bam file (22).

### H&E and Immunohistochemistry Staining

The formalin-fixed and paraffin-embedded tissue was cut into 4-μm thick sections and affixed onto the slides. The slides were subjected to H&E staining and immunohistochemistry. After being deparaffinized and rehydrated, the antigens were retrieved in boiled Tris-EDTA (pH 9.0) buffer for 15 min, cooled off for 1 h in the fume hood, and then blocked according to the protocol of the DAB polymer detection kit (Gene Tech, Shanghai, China) for 10 min. The slides were incubated with primary antibody in 1% bovine serum albumin (BSA)/tris-base solution buffer at 4°C overnight. The next day, the slides were incubated with the secondary antibody and developed with DAB reagent according to the protocol of the DAB polymer detection kit (Gene Tech). Finally, the slides were counterstained with hematoxylin. Anti-CD3 antibody (Catalog Number : AR0042, Talent Biomedical, 1:500), anti-CD4 antibody (Catalog Number : AR0273, Talent Biomedical, 1:500), anti-CD8 antibody (Catalog Number : AM0063, Talent Biomedical, 1:500), anti-TIA-1 antibody (Catalog Number : AM0226, Talent Biomedical, 1:500), anti-Granzyme B antibody (Catalog Number : AM0308, Talent Biomedical, 1:500), anti-Perforin antibody (Catalog Number : AM0311, Talent Biomedical, 1:500), anti-Ki67 antibody (Catalog Number : AR0248, Talent Biomedical, 1:500), anti-CXCL13 (Catalog Number:10927-1-AP, Proteintech, 1:500), anti-TIMD4 (Catalog Number:12008-1-AP, Proteintech, 1:500), and anti-VCAM1 (Catalog Number:11444-1-AP, Proteintech, 1:400) were used.

## Results

### Clinical Characteristics of the Studied Patient With SPTCL

The clinical manifestations of the studied patient with SPTCL are summarized in **Table 1**. The initial disease onset of this male patient was at six months old, diagnosed with a small hard nodule (diameter: 1 cm) in the left clavicle and enlarged lymph nodes in the groin. When he was 12 months old after a measles vaccination, the initial nodule was significantly enlarged (diameter: 6 cm) with enlarged lymph nodes in the head of the pancreas and did not decrease significantly after antibiotic treatment, puncture, and drainage. At the age of 18 months, the disease progressed with multiple lesions at the root of the patient’s right thigh (diameter: 5 cm) accompanied by fever and then at the left hip (diameter: 3 cm) after anti-inflammatory treatment for controlling body temperature. Four months later (22 months old), the patient progressed with a new single lesion at the right shoulder (diameter: 2 cm) with no fever but enlarged lymph nodes in the neck, underarms, mediastinum, and groin. Subcutaneous lesions were more common in the extremities and partly in the trunk. The lesions varied from 1 cm to 6 cm in diameter, with redness and swelling. No ulcerated plaque was observed. Multiple lymphadenopathies were proven by computerized tomography (CT) scans without hepatomegaly. The patient did not receive any chemotherapy but was followed up according to his parents’ decision.

**Table 1:** The clinical characteristics of multiple episodes in the patient with SPTCL analyzed in this study.

The onset of symptoms (months)	Subcutaneous lesions		Other lesions	Systemic symptoms	HLH	EBV infection	Histopathological characteristics			Immunophenotype		TCR gene rearrangement
	Location	Diameter					Infiltration range	Infiltration cell	Types	IHC	Level of Ki‐67	
**6**	Single lesion: left clavicle	1cm	Enlarged lymph nodes of the groin	None	No	No	NA	NA	NA	NA	NA	NA
**12**	Single lesion: left clavicle	6cm	Enlarged lymph nodes in the head of the pancreas	None	No	No	Dermis and subcutaneous tissue	Lymphocytes and histocytes	Inflammation	CD1α (-), CD34 (+), CD45 (+), CD68 (+)	5%	NA
**18**	Multiple lesions: right thigh and left hip	3-5cm	Enlarged lymph nodes of the neck, underarms, mediastinum, and groin	Fever	No	No	Epidermis, dermis and subcutaneous fatty tissue	Heterotypic lymphocytes, histocytes and karyokinesis	Panniculitis-like	CD20 (-), CD3 (+), CD5 (+), CD7 (+), CD4 (-), CD8 (+), TIA (+), GB (+/-), CD56 (-), EBER (-)	40-50%	NA
**22**	Single lesion: right shoulder	2cm	Enlarged lymph nodes of the neck, underarms, mediastinum, and groin	None	No	No	Dermis and subcutaneous fatty tissue	Heterotypic lymphocytes, histocytes, karyokinesis and karyorrhexis	Panniculitis-like	CD20 (-), CD3 (+), CD5 (+), CD7 (+), CD4 (+/-), CD8 (+), TIA (+/-), GB (+/-), Perforin (+/-), CD56 (-), EBER (-)	20%	Clonal TCR-Beta gene rearrangements

EBV, Epstein-Barr virus; HLH, hemophagocytic lymphohistiocytosis; SPTCL, subcutaneous panniculitis-like T-cell lymphoma.

**Figure 1:**
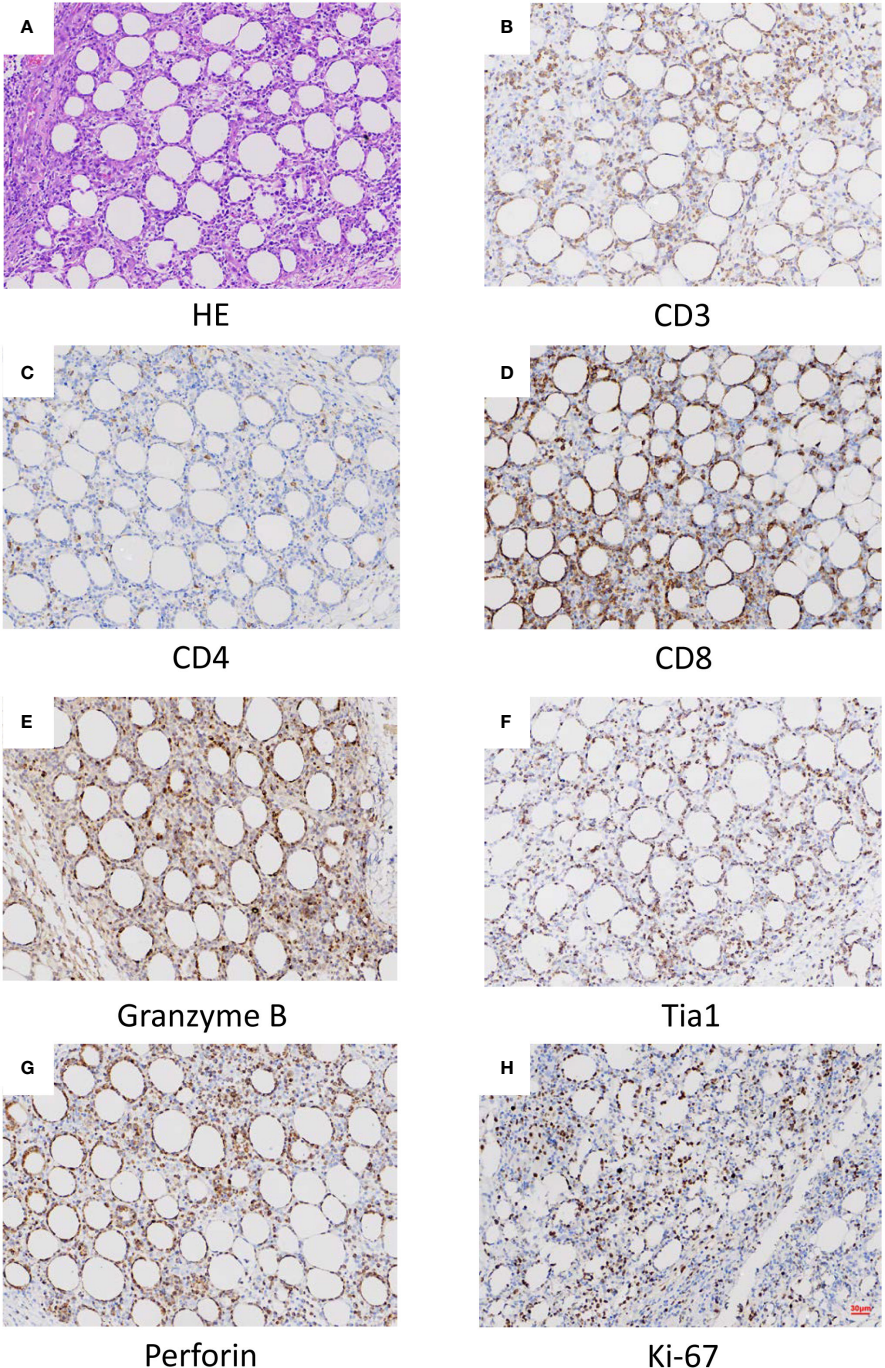
Histopathological **(A)** and histochemical **(B–H)** results of the lesion. **(A)** Sections at low power stained with hematoxylin and eosin showing a heavy lymphocytic infiltrate predominantly in the subcutis (x40). **(B)** CD3 positive (x40). **(C)** CD4 in approximately 5% of cells (x40). **(D)** CD8 positive (x40). **(E)** Granzyme B positive (x40). **(F)** Tia1 positive (x40). **(G)** Perforin positive (x40). **(H)** Ki-67 positive (x40).

Histopathological, immunophenotypical, and molecular features of the patient samples are also summarized in **Table 1**. All skin biopsy specimens demonstrated a dense lymphoid infiltrate located in the subcutaneous tissue, with the overlying epidermis and dermis involved. Atypical lymphocytes were pleomorphic small to medium-sized to diffusely large T cells with irregular hyperchromatic nuclei and were admixed with small lymphocytes and histiocytes, which were found in both biopsy specimens at 18 and 22 months old. Areas of karyokinesis and karyorrhexis were seen. These atypical lymphocytes showed a CD3^+^, CD4^–^, CD8^+^, Granzyme B^+^, Perforin^+^, and TIA1^+^ phenotype (**Figure 1**) with a high proliferation rate. Epstein-Barr virus (EBV) detection by EBV-encoded RNA (EBER) in situ hybridization was negative. Clonal rearrangement of the TCR beta gene was found in the biopsy at 22 months old. In all episodes, bone marrow examination showed no evidence of lymphoma.

### The SPTCL-Specific Ecosystem at Single-Cell Resolution

**Figure 2:**
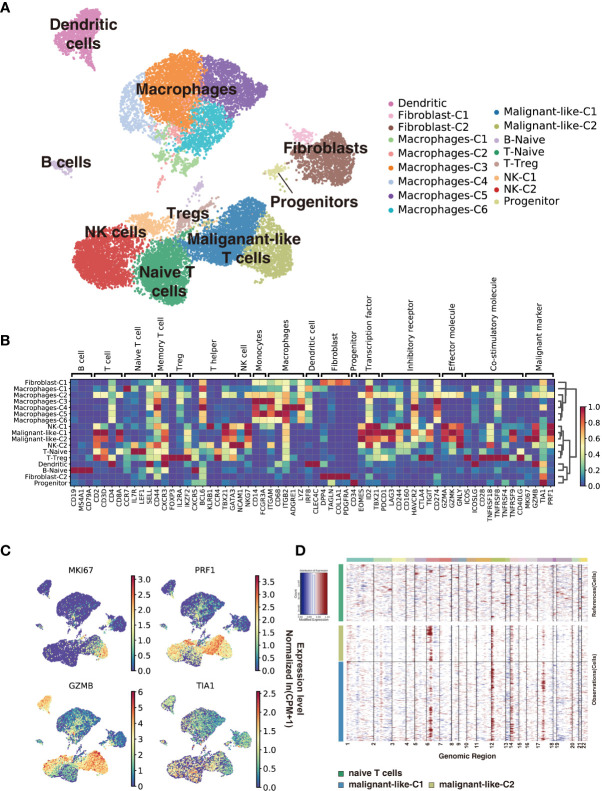
Subcutaneous panniculitis-like T-cell lymphoma ecosystem at single-cell resolution. Cells from the patient’s subcutaneous lesion tissue were clustered using the Leiden community detection algorithm to identify groups of cells with similar expression patterns. **(A)** Single-cell expression of the subcutaneous lesions’ cells in UMAP space (first two dimensions). Cells are color-coded according to the clusters generated by the Leiden algorithm. **(B)** Heatmap summarizes the mean expression (normalized and log-transformed) of selected canonical markers in each cluster. The gene expression value has been scaled for visualization. The covariate bar on the top side indicates the component associated with each gene, and red boxes highlight the prominent expression of genes for the known subtypes. **(C)** UMAP plots of malignant markers (*MKI67, PRF1, TIA1, GZMB*) expression in subcutaneous lesions’ cells. **(D)** Chromosomal landscape of inferred large-scale copy number variations (CNVs) distinguishes malignant from non-malignant cells. Amplifications (red) or deletions (blue) were inferred by averaging expression over 100-gene stretches on the respective chromosomes.

We used scRNA-seq to profile gene expression in cells obtained from the enzymatically digested subcutaneous lesion tissue of the biopsy before any treatment at 22 months old. Transcriptomic data were obtained from a total of 17,598 cells, with a median of 1,672 genes detected per cell. Cells were grouped according to their expression profiles by principal component analysis (PCA) and Uniform Manifold Approximation and Projection (UMAP) dimensional reduction. Unsupervised graph-based Leiden clustering by *Scanpy* identified 17 clusters of cells that were annotated and assigned with a cell type based on the expression of genes described in known canonical markers and published transcriptome data (see Methods for details) (**Figures 2A, B** and **Supplementary Figure S1**). These included one naïve T cell cluster, one Treg cell cluster, two CD8^+^ T cell clusters, two NK cell clusters, one naïve B cell cluster, six macrophage clusters, one dendritic cell cluster, two fibroblast clusters, and one progenitor cell cluster. Macrophages were the most abundant immune cells in our study, with a low proportion of B cells. Malignant-like T cells were identified based on conventional SPTCL markers (i.e., *MKI67*, *PRF1, TIA1*, and *GZMB*; **Figure 2C**), which were highly expressed in these two CD8^+^ T cell clusters. However, we cannot rule out the possibility that there were a few normal CD8^+^ T cells in these two clusters since some markers such as *GZMB* and *PRF1* were also expressed to a certain extent in normal CD8^+^ T cells. To validate the identification of malignant-like T cells, we further distinguished malignant from non-malignant T cells by inferring large-scale chromosomal copy-number variations (CNVs) based on transcriptomes (**Figure 2D**). As expected, almost all the identified malignant-like cells (>99%) showed clear evidence of a gain of 6p, 12p, and 14p compared with normal reference cells, supporting that most of them were real malignant cells.

Next, we use cNMF (15) to infer potential GEPs underlying the expression profiles and which cells expressed the GEPs. We identified 23 distinct programs in this dataset, which were further divided into identity programs (n=19) and activity programs (n=4) based on the criterion that the former represents a unique cell type while the latter can occur in multiple diverse cell types (**Supplementary Table S1**; **Supplementary Figure S2A**). Most cells had only one GEP, which represents their identity program. In addition to the 17 primary cell-type clusters initially generated by Scanpy and refined by the identity GEPs, we also identified epithelial cells, endothelial cells, and mast cells in this SPTCL-specific ecosystem (**Supplementary Figure S2A**). We noticed that the identified malignant T cells expressed one identity GEP that was significantly enriched for genes involved in cell killing and T cells activation (**Supplementary Figures S2A, B**), including the chemokines family *(i.e., CCL5, CCR5, CXCR3, CXCR6)*, cytotoxic proteins *(i.e., NKG7, GZMA, GZMB, GZMH, GZMK, GNLY, PRF1)*, and immune checkpoint genes *(i.e., LAG3, CD27, TIGIT, HAVCR2, PDCD1, CTLA4)* (**Supplementary Figure S2C**; **Supplementary Table S1**). Some malignant T cells also expressed an activity GEP named Proliferation, which was strongly enriched for genes associated with cell cycle (e.g., Mitotic Nuclear Division; **Supplementary Figures S2A, B**). Moreover, parts of malignant T cells expressed an activity GEP named Act.T, which was also expressed in naïve T cells and NK cells (**Supplementary Figure S2A**).

### Comparison of Malignant and Normal T Cells by Expression Profiling

**Figure 3:**
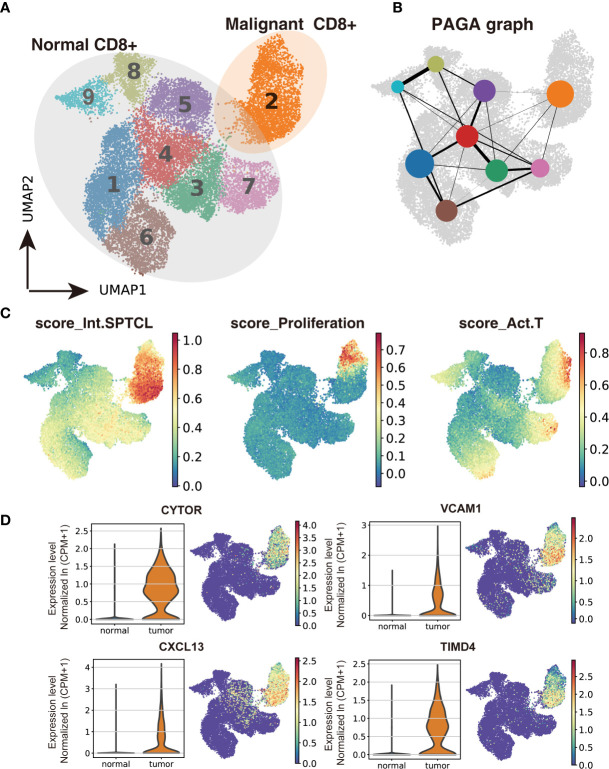
Transcriptomic comparison of malignant versus normal CD8^+^ T cells. **(A)** UMAP projection of cells from healthy donors and the patient’s subcutaneous lesion tissue with normal CD8^+^ T cells outlined in grey and malignant CD8^+^ T cells in orange. **(B)** Results of partition-based graph abstraction (*PAGA*). Each node represents a cluster, and edges show the connectivity between clusters. The size of nodes indicates the number of cells in each cluster, and the edge thickness shows the connection strength. **(C)** Results of GEP-program cell scoring in UMAP space (first two dimensions). **(D)** Potential novel markers of SPTCL cells with a Δ percentage of cells expressed greater than 90% and *P*_adj_ < 1e-100. CPM, counts per million.

To investigate the difference between malignant and normal T cells, we paired isolated T cells from the subcutaneous lesion tissue of the patient with normal T cells from donors’ peripheral blood, bone marrow, lung tissues, and lymph nodes. We applied Scanorama to correct the potential batch effects between two datasets and merged the neighbor sets *via* the UMAP algorithm as a combined dataset. Based on the cell-type clusters in SPTCL, we found that the naïve T cells from the patient overlapped with normal T cells from donors, while malignant T cells were obviously separated from them (**Supplementary Figure S3**). Using the Leiden clustering algorithm, we identified nine UMAP clusters presenting the normal versus malignant classification clearly (**Figure 3A**), actively supporting the separation within the UMAP. The graph-like maps of cells generated by the partition-based graph abstraction (PAGA) also confirmed these two distinct populations without secure connections (**Figure 3B**).

As reported by Gayden et al. (8), germline *HAVCR2* mutations altering TIM-3 were significantly overrepresented in SPTCL patients, especially with hemophagocytic lymphohistiocytosis (HLH). They also observed elevated serum levels of IFN-γ-induced CXCL10, inflammasome-activated interleukin-18 (IL-18), and soluble CD25 in a *HAVCR2* mutant SPTCL patient at the time of active disease, and increased amounts of tumor necrosis factor-α (TNF- α) and IL-2 produced *in vitro* by T lymphoblasts from *HAVCR2* mutant patients with SPTCL. Thus, we checked the genotype of *HAVCR2* by examining the whole-exome sequencing (WES) data of the patient’s SPTCL tissue and did not observe any coding mutation in the *HAVCR2* gene (**Supplementary Figure S4**). We also checked the expression of genes (*HAVCR2, TNF, IL2, CXCL10, IL18*, and *IL2RA/CD25*) in our scRNA-seq data for malignant and normal T cells and found regular expression of *HAVCR2* and *CXCL10* and low expression of *TNF*, *IL2, IL18*, and *IL2RA(CD25)* in the SPTCL malignant cells (**Supplementary Figure S5**) compared with normal T cells, suggesting the difference between *HAVCR2*-wild-type and mutant SPTCL patients.

It has previously been proposed that regulatory T lymphocytes (Treg) could play an essential role in SPTCL pathogenesis, especially in the skin (8, 23). In particular, Gayden et al. identified a drastic decrease in FOXP3^+^CD4^+^ T cells in TIM-3 mutants compared with TIM-3 wild-type SPTCL (8). For comparison, we isolated FOXP3^+^CD4^+^ T cells in our SPTCL scRNA-seq data and found that the proportion (21.97%) of FOXP3^+^CD4^+^ T cells in CD4^+^ T cells in our patient was similar to that in TIM-3 wild-type SPTCLs and higher than that in TIM-3 mutants in the reported cohort, consistent with their finding (**Supplementary Figure S6**).

Next, we used the conventional SPTCL markers to examine the separation of normal and malignant cells above. As expected, we found that the classical SPTCL marker *MKI67* was very specifically observed in malignant cells but mostly not seen in normal cells, while the markers *GZMB* and *PRF1* were not only expressed in the tumor T cells but also in part of normal CD8^+^ T cells (**Supplementary Figure S7**). Then we scored each cell by their gene expression correlation to malignant GEPs, including the previously identified malignant identity GEP (Int.SPTCL) and activity GEPs (Proliferation and Act.T). There were significant differences in malignant and normal T cells scored with all these GEPs (P< 0.001) (**Figure 3C**).

Meanwhile, we also paired the isolated T cells from the subcutaneous lesion tissue with T cells from the peripheral blood and bone marrow of the patient using the same process. Interestingly, there were a small amount of CD8^+^ T cells from peripheral blood and bone marrow in proximity to malignant T cells, and PAGA analysis also showed connections between them (**Supplementary Figures S8A, B**). Furthermore, we also found that the malignant T cells and the proximate T cells from matched peripheral blood and bone marrow were scored significantly higher than others with GEPs named Int.SPTCL and Proliferation (**Supplementary Figure S9**), suggesting that malignant-like or pre-malignant cells may exist in the circulation of the patient resulting in malignant recurrence.

To identify potential novel markers and/or therapeutic targets of SPTCL, we performed differential gene analysis by comparing the malignant cells to normal T cells. In total, we identified 45 significantly overexpressed genes in the malignant cells as potential markers for SPTCL (*P*_adj_ < 0.05 and average fold change >2). As expected, the top upregulated genes in the malignant cells were *GNLY* and the granzyme subfamily (e.g., *GZMA, GZMK*) (**Supplementary Figure S10**). We further identified potential novel markers for SPTCL including *CYTOR, CXCL13, VCAM1*, and *TIMD4*, which were specifically differentially expressed in the SPTCL cells as defined by average fold change > 2 and delta percentage > 90% in malignant T cells versus normal T cells (**Figure 3D**). Moreover, we also examined previously reported SPTCL-related genes (10) and found a group of genes significantly differentially expressed in the SPTCL cells (i.e., *APOBEC3G, CCL4, CCL5, CXCL10, CXCR3, FASLG, GBP5, IFNG, IKZF3, KLRD1, PRF1, and TNFRSF9* (*P*_adj_ < 1 x 10^-10^; **Supplementary Figure S11**). The complete results for differential expression analysis are included in **Supplementary Table S2**.

Next, we focused on three of these potential novel markers, *CXCL13*, *VCAM1, and TIMD4*, which are protein-coding genes and presented no or shallow expression in normal lymphocytes. Their expression was examined by immunohistochemistry in the patient’s subcutaneous lesion and additional samples from patients with panniculitis (PA) (**Supplementary Figure S12**). Results showed that PA lesions were negative or weakly positive for the expression of these markers, while SPTCL lesions exhibited high numbers of positive cells for all three markers.

### Single-Cell Expression Patterns of Novel SPTCL-Specific Immune Subsets

**Figure 4:**
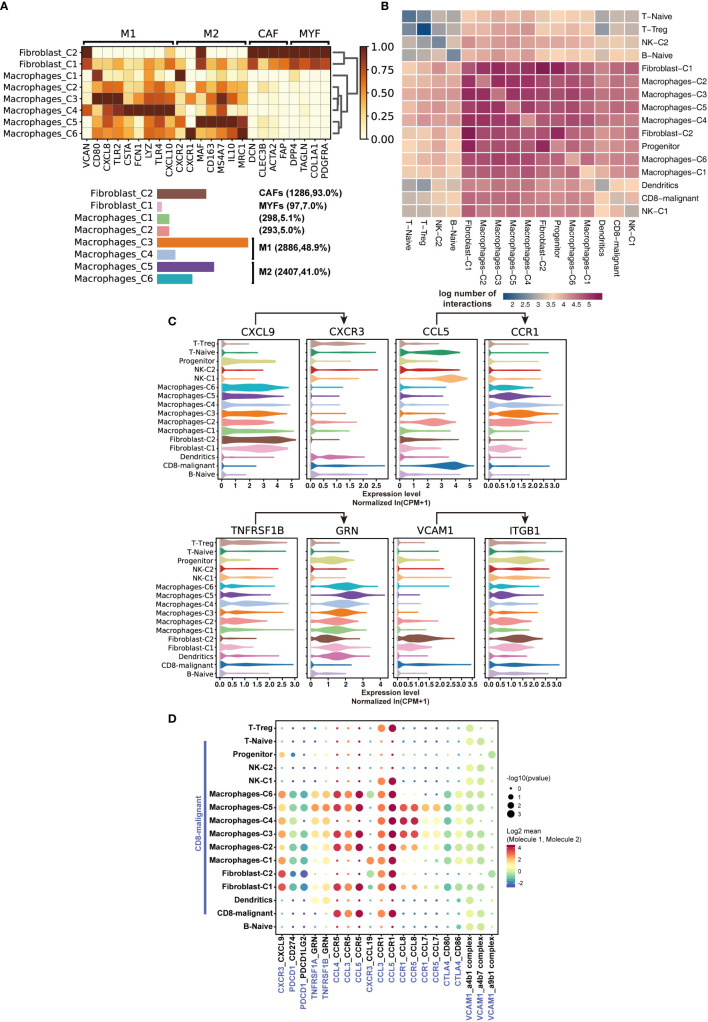
Characteristics of SPTCL-specific immune subsets. **(A)** Heatmap summarizing mean expression (normalized and log-transformed) of M1, M2, CAF, and MYF markers in each cluster (above). Bar plot showing the cell fraction of subsets of macrophages and fibroblasts (below). M1, classically activated macrophage; M2, alternatively activated macrophage; CAF, cancer-associated fibroblasts; MYF, myofibroblasts. **(B)** Heatmap depicting the log number of all possible interactions between the clusters analyzed. **(C)** Violin plots showing expression of ligands *CXCR3, CCL5, TNFRSF1B*, and *VCAM1* and cognate receptors *CXCL9, CCR1, GRN*, and *IGTB1* on respective stromal populations. **(D)** Dot plot depicting selected tumor-immune interactions enriched in the microenvironments.

To further characterize immune cells in the tumor environment of SPTCL, we annotated and dissected macrophages and fibroblasts based on the expression of genes described in known canonical markers (**Figure 4A**). We found that most of the macrophages were of the M1-type (classically activated macrophage) and M2-type (alternatively activated macrophage) with similar proportions (48.9% vs. 41.1%). The other two clusters of macrophages were not in polarized activation states; thus, they may be in M0 resting states. Intriguingly, we also identified a group of cancer-associated fibroblasts (CAFs), a type of perpetually activated fibroblasts, based on the “CAF markers”, suggesting that these cells could emerge as players in immune regulation.

Next, we sought to elucidate the interactions of the malignant T cells with the immune populations by examining the cross-talk between them. We systematically predicted cell-cell communication networks based on *CellPhoneDB* (19), a manually curated repository of ligands, receptors, and their interactions integrated with a statistical framework to infer cell-cell communication networks from single-cell transcriptomic data. We found that the interactions of malignant T cells occurred more frequently with macrophages, fibroblasts, and dendritic cells, compared with naïve T cells, Treg, NK cells, and B cells (**Figures 4B, C**). Notably, macrophages and fibroblasts dominated the cell-cell communication landscape in this microenvironment, suggesting they might play the primary role in tumor-immune interactions of SPTCL. There was no significant difference between M1 and M2 macrophages in tumor-immune interactions. We identified multiple tumor-immune interactions, for example between *CXCR3, CCL5, TNFRSF1B*, and *VCAM1*-expressing malignant T cells and macrophages/fibroblasts positive for *CXCL9, CCR1, GRN*, and *IGTB1*, respectively (**Figure 4C**). Interestingly, we found that the recruited macrophages might promote the inflammatory activity of malignant T cells *via* suppressing the *PDCD1* and *CTLA4* axis (**Figure 4D**), because the *PDCD1*(PD-1)-*CD274*(PD-L1) and *CTL4-CD80/86* interactions can inhibit activation, expansion, and acquisition of effector functions of CD8^+^ T cells (24).

## Discussion

SPTCL is a rare disease facing significant diagnostic challenges. The clinical manifestations of SPTCL are complex with only a few consistent characteristics. Subcutaneous tissue infiltration and/or infiltration by CD3^+^CD8^+^ cells expressing cytotoxic proteins (GZMB, TIA-1, perforin) is the typical pathological change of SPTCL (25). However, this change can also occur in benign panniculitis and lupus erythematosus profundus caused by autoimmune attacks (26, 27). The patient in this study experienced multiple subcutaneous mass in 16 months, and the results of the biopsy have shown that the mass evolved from benign to malignant. Because no standardized therapeutic approach has been established for SPTCL, the patient received two surgeries to remove the tumor without chemotherapy or radiotherapy. Interestingly, after the final operation, the patient has been followed up for more than one year and has not suffered a relapse. Thus, if there are proper approaches to effectively diagnose the disease and specific markers to distinguish malignant cells of SPTCL, timely surgical resection could be an effective therapy.

Here, we used scRNA-seq profiling of the malignant and normal cells from the SPTCL patient and normal cells from healthy donors to characterize the molecular events of SPTCL. To our knowledge, this is the first study exploring gene expression signatures, summarizing the tumor microenvironment of SPTCL in single-cell resolution. We identified a unique GEP that was expressed significantly higher in SPTCL cells than in normal T cells, which could be a characteristic of SPTCL. We found four genes (i.e., *CYTOR, CXCL13, VCAM1*, and *TIMD4*) explicitly expressed in malignant T cells, which may be potential novel markers for SPTCL. We also investigated interplays between different stromal populations and malignant T cells and found the leading role of macrophages and fibroblasts (especially CAFs) in the SPTCL microenvironment, suggesting their contribution to malignant T cell dysfunction. More specifically, the recruited macrophages might suppress the *PDCD1* and *CTLA4* axis to enhance the inflammatory activity of malignant cells, consistent with the clinical manifestation of SPTCL.

*CYTOR* (or Linc00152) is a long non-coding RNA that is overexpressed in multiple cancer cells, and it can promote cell proliferation and epithelial-mesenchymal transition (28). Given its crucial role in the pathogenesis of cancers, *CYTOR* (average fold change = 3.40, Δ percentage =95%) may play a role in SPTCL development.

*CXCL13*, initially identified as a B-cell chemoattractant, exerts essential functions in lymphoid neogenesis and has been widely implicated in the pathogenesis of several autoimmune diseases and inflammatory conditions, as well as in lymphoproliferative disorders (29). This chemokine has been proposed as a marker for certain lymphomas, such as angioimmunoblastic T-cell lymphoma (AITL), an aggressive nodal T-cell lymphoma derived from T_FH_ cells (2, 30). The SPTCL malignant cells highly expressed *CXCL13*, suggesting that its role in SPTCL is intriguing and worth exploring.

Vascular adhesion molecule-1 (*VCAM1*), a member of the immunoglobulin family of cell-cell adhesion receptors, is expressed aberrantly in some tumor cells, such as renal, breast, or gastric carcinomas (31–33). Clustering of VCAM-1 on the cell surface, acting through Ezrin, triggers Akt activation and protects cancer cells from proapoptotic cytokines such as the TNF-related apoptosis-inducing ligand (TRAIL) (32, 34). VCAM-1 can tether macrophages to cancer cells *via* counter-receptor α4β1-integrins, and we found that macrophages and fibroblasts in the SPTCL microenvironment highly expressed *ITGA4* and *ITGB1*, which constitute α4β1-integrins. The interaction between malignant T cells and immune cells may possess similar effects like *VCAM1*-mediated mechanisms in breast cancer cells (32, 34).

*TIMD4*, a member of the TIM family of immunoregulatory proteins, is overexpressed in multiple tumor tissues, which has been proven to promote tumor cell growth and proliferation both *in vitro* and *in vivo* in lung cancer (35). As reported in recent studies, *TIMD4* is expressed in professional antigen-presenting cells (APCs), pro-B cells (36), and NKT cells (37) but not in normal CD8^+^ T cells. Its role of aberrant expression in SPTCL cells needs to be explored further in the future.

Single-cell methods allow researchers to characterize the tumor transcriptome and microenvironment in an unprecedented resolution. Our study offered a new insight into the heterogeneity of subcutaneous panniculitis-like T-cell lymphoma, providing a better understanding of the transcription characteristics and immune microenvironment of this rare tumor. This new level of data provided an opportunity for clinically meaningful advances in SPTCL.

## Data Availability Statement

The datasets presented in this study can be found in online repositories. The names of the repository/repositories and accession number(s) can be found below: the Genome Sequence Archive (GSA) for Human in National Genomics Data Center (NGDC) under accession number HRA000370.

## Ethics Statement

The studies involving human participants were reviewed and approved by Children’s Hospital of Fudan University Research Ethics Board. Written informed consent to participate in this study was provided by the participants’ legal guardian/next of kin.

## Author Contributions

XWZ and MQ are the principal investigators of this study and take responsibility for the integrity of the data and the accuracy of the data analysis. ZL analyzed the single-cell RNA sequencing data. XWZ, RD, MQ, and ZL wrote the manuscript. HW, JM, LS, XQ, XHZ, PC, HM, YY, YF, PW, WJ, CS, YM, CL, XY, SJ, HZ, and RD provided advice on the study design and participated in data collection. XWZ, RD, MQ, and ZL interpreted the data and the research findings. All authors contributed to the article and approved the submitted version.

## Funding

The work was supported by the Health and Family Planning Commission of Shanghai Municipality (201740011), the National Natural Science Foundation of China (81973997), and the Cyrus Tang Foundation. MQ is supported by the Program for Professor of Special Appointment (Eastern Scholar) at Shanghai Institutions of Higher Learning.

## Conflict of Interest

The authors declare that the research was conducted in the absence of any commercial or financial relationships that could be construed as a potential conflict of interest.
