# Supplementary material for: Single-Cell RNA-seq Reveals Characteristics of Malignant Cells and Immune Microenvironment in Subcutaneous Panniculitis-Like T-Cell Lymphoma
Source: Front Oncol. 2021 Mar 18;11:611580. doi: 10.3389/fonc.2021.611580 (PMC8013729; doi:10.3389/fonc.2021.611580)
Supplement: Supplementary file 1 [file DataSheet_1.pdf]

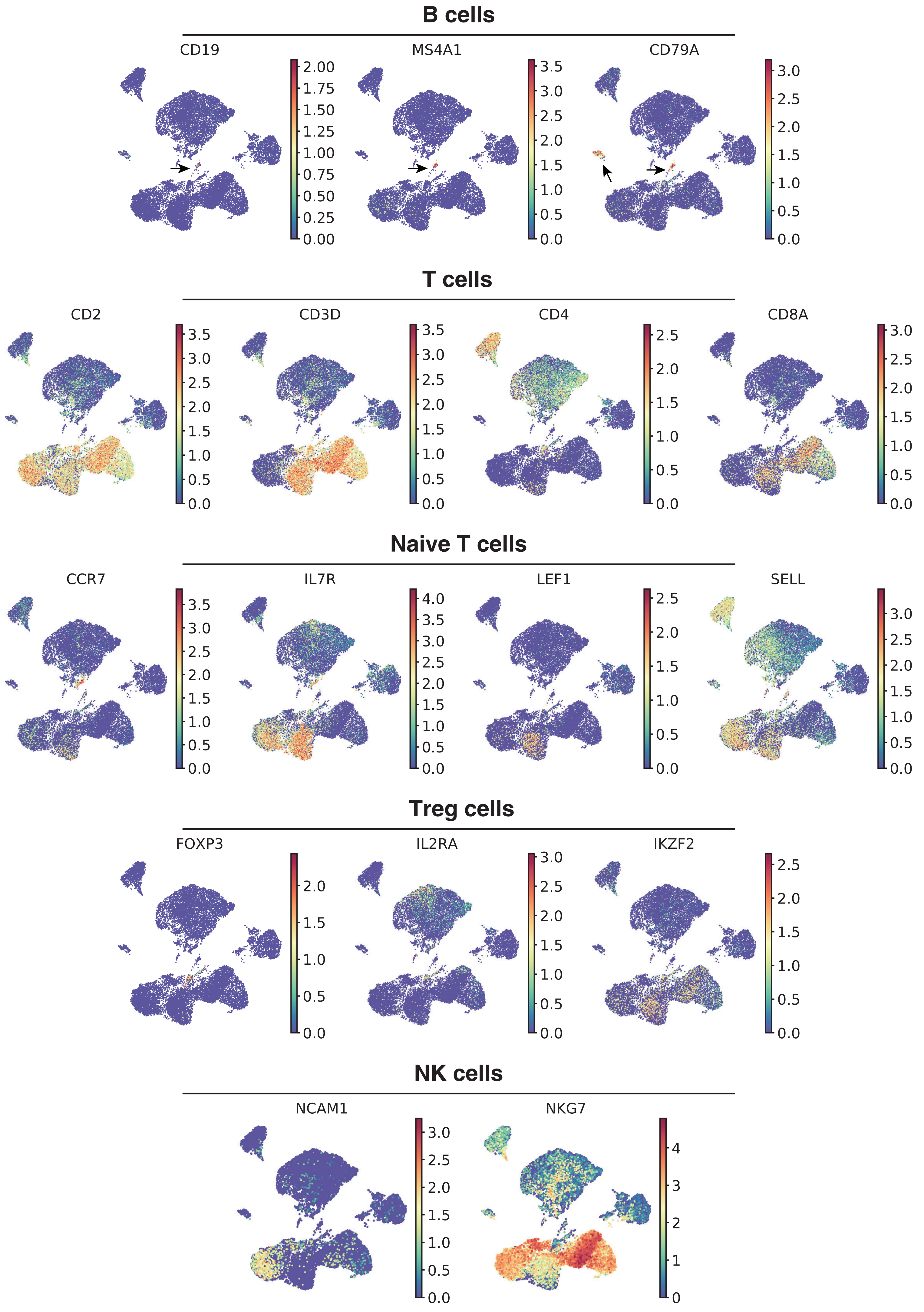

B cells
CD19
MS4A1
CD79A
T cells
CD2
CD3D
CD4
CD8A
Naive T cells
CCR7
IL7R
LEF1
SELL
Treg cells
FOXP3
IL2RA
IKZF2
NK cells
NCAM1
NKG7

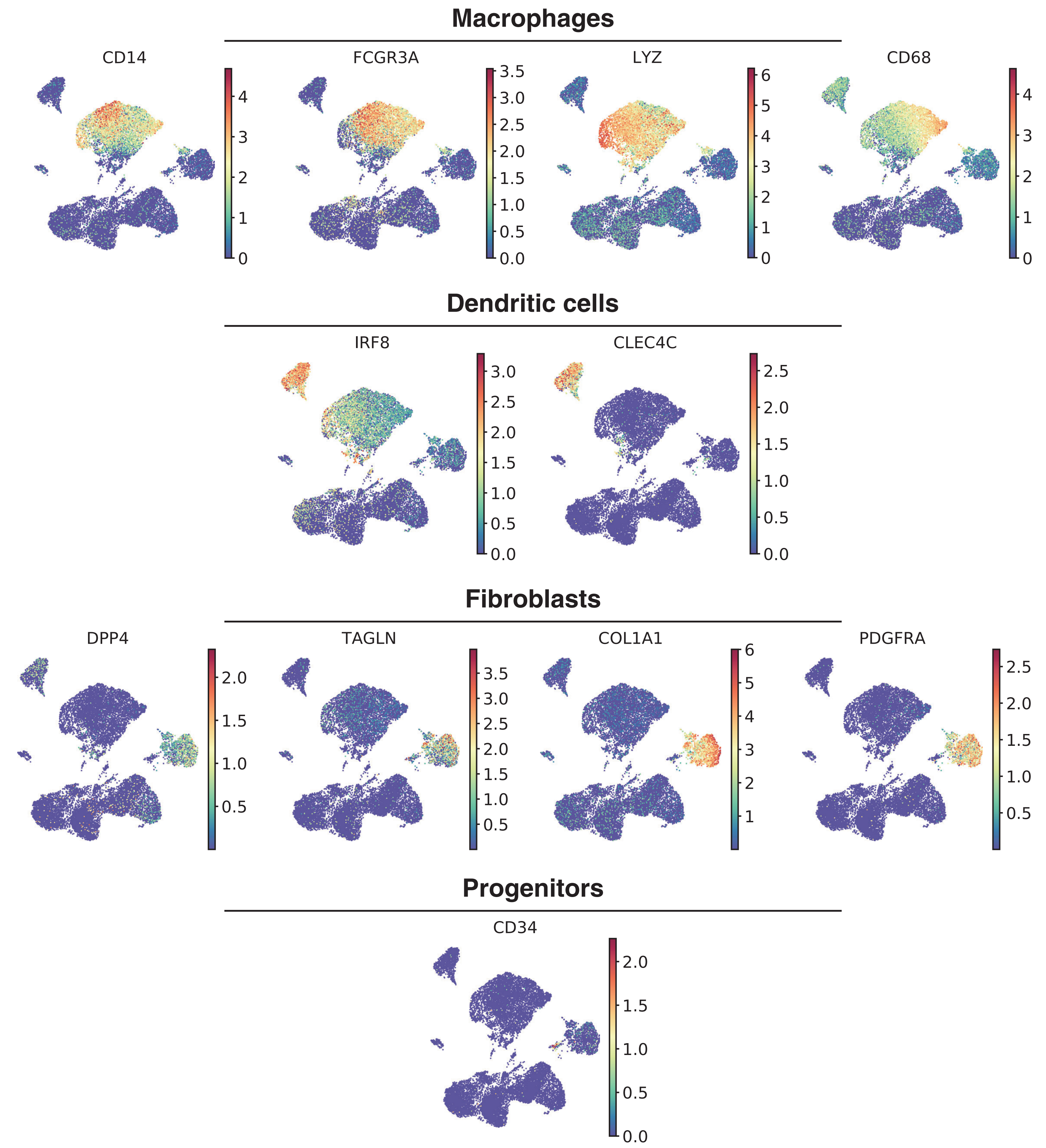

Supplemental Figure S1. The canonical marker genes expression for cell-type annotation of cells from the patient's subcutaneous lesion, related to Figure 2A-B.

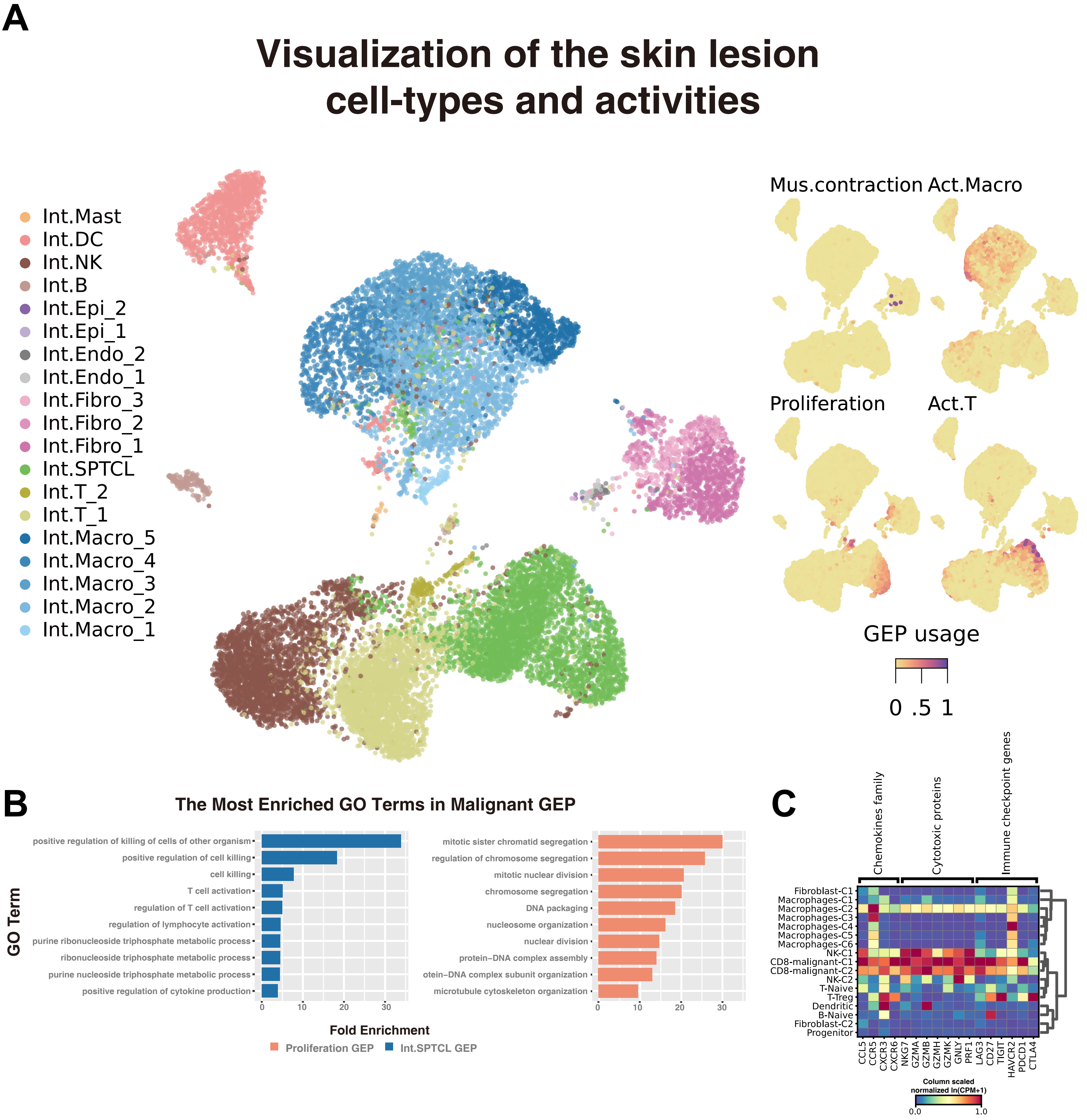

Supplemental Figure S2. Identification of activity programs and identity programs in the tumor tissue. (A) UMAP plots of cells colored by maximum identity GEP usage (left) or by absolute usage of each activity GEP (right). Int, identity; DC, dendritic cells; NK, nature killer cells; B, B cells; Epi, epithelial cells; Endo, endothelia cells; Fibro, fibroblasts; SPTCL, Subcutaneous panniculitis-like T-cell lymphoma; T, T cells; Macro, macrophages; Mus, muscle. **(B)** Bar plots of P-values for the top 10 Gene Ontology geneset enrichments for the identity and activity GEPs.(C) Heatmap summarizing mean expression (normalized and log-transformed) of Int.SPTCL markers in each cluster. The gene expression value has been scaled for visualizations. The covariate bar on the top side indicates the component associated with each gene.

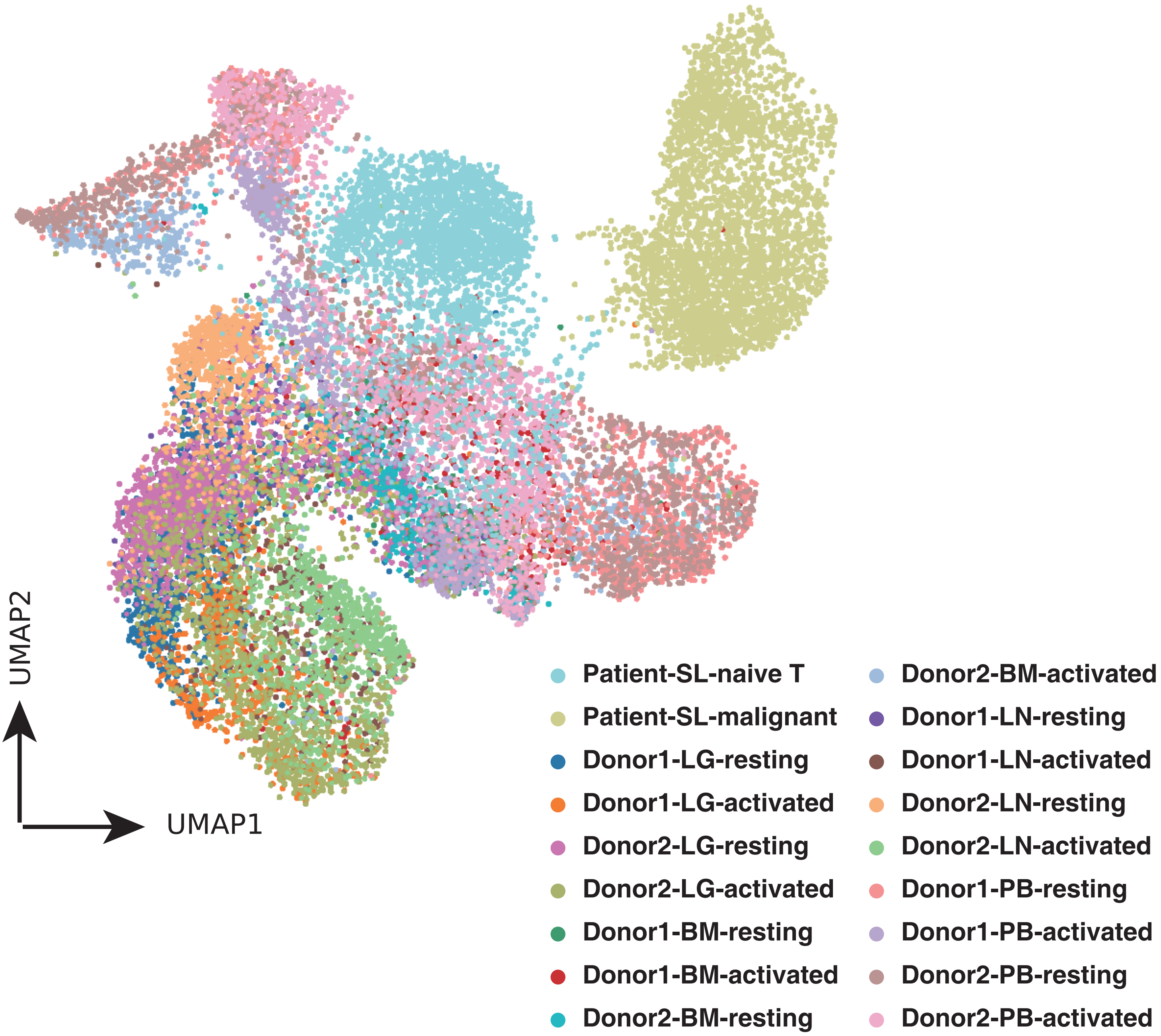

Supplemental Figure S3. UMAP projection of donors' samples with normal $CD8^+$ T cells (n=13,494) and patient sample with $CD8^+$ cells (n=5,956) in subcutaneous lesion tissue, related to Figure 2. BM, bone marrow; LG, lung; LN, lymph node; PB, peripheral blood; resting; SL, subcutaneous lesion; $CD3^+$ T cells isolated from tissues and blood were cultured in media alone; activated, in the presence of anti-CD3/anti-CD28 antibodies.

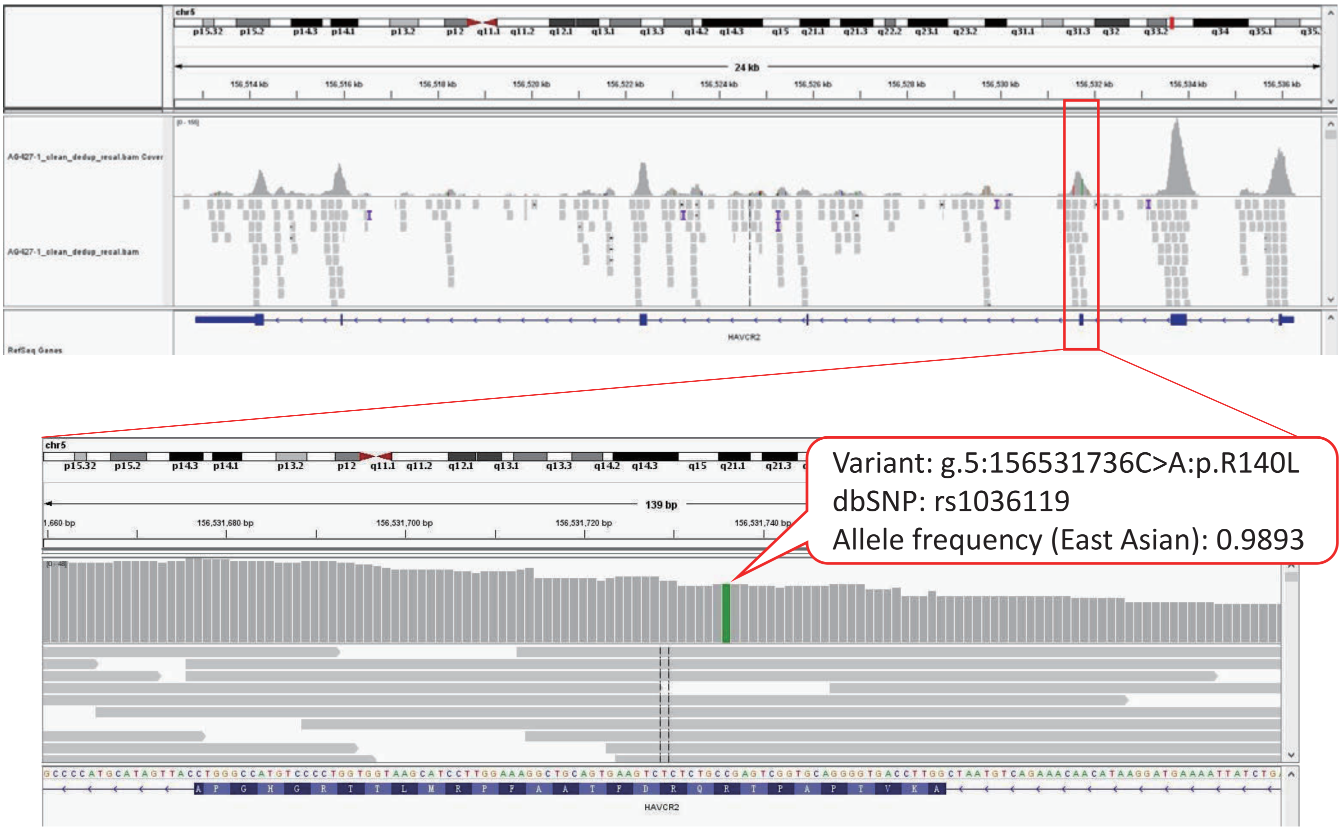

Supplemental Figure S4. Integrative genomics view of the *HAVCR2* genotype with whole-exome sequencing in the patient's subcutaneous lesion tissue. The result showed that there was no coding mutation existed in the *HAVCR2* gene, except for a common coding SNP (p.R140L, rs1036119) with allele frequency 0.9893 in East Asian population based on the genome aggregation database (gnomAD v2.1.1).

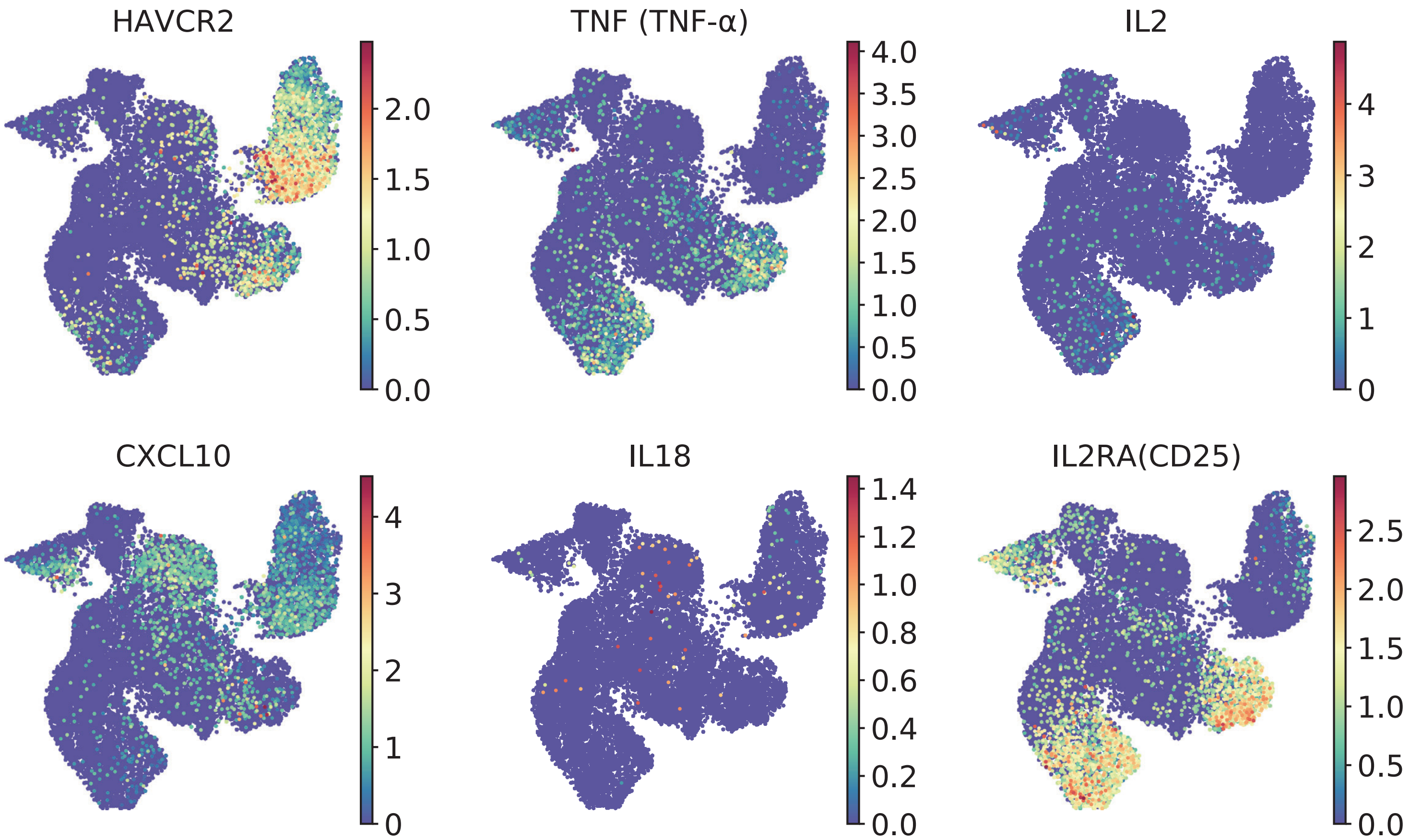

Supplemental Figure S5. UMAP embedding of three gene expression (*HAVCR2, TNF, IL2, CXCL10, IL18, IL2RA*). The results showed that the SPTCL malignant cells with no mutations of *HAVCR2* had regular expression of *HAVCR2* and *CXCL10* and low expression of *TNF, IL2, IL18*, and *IL2RA* compared with normal T cells.

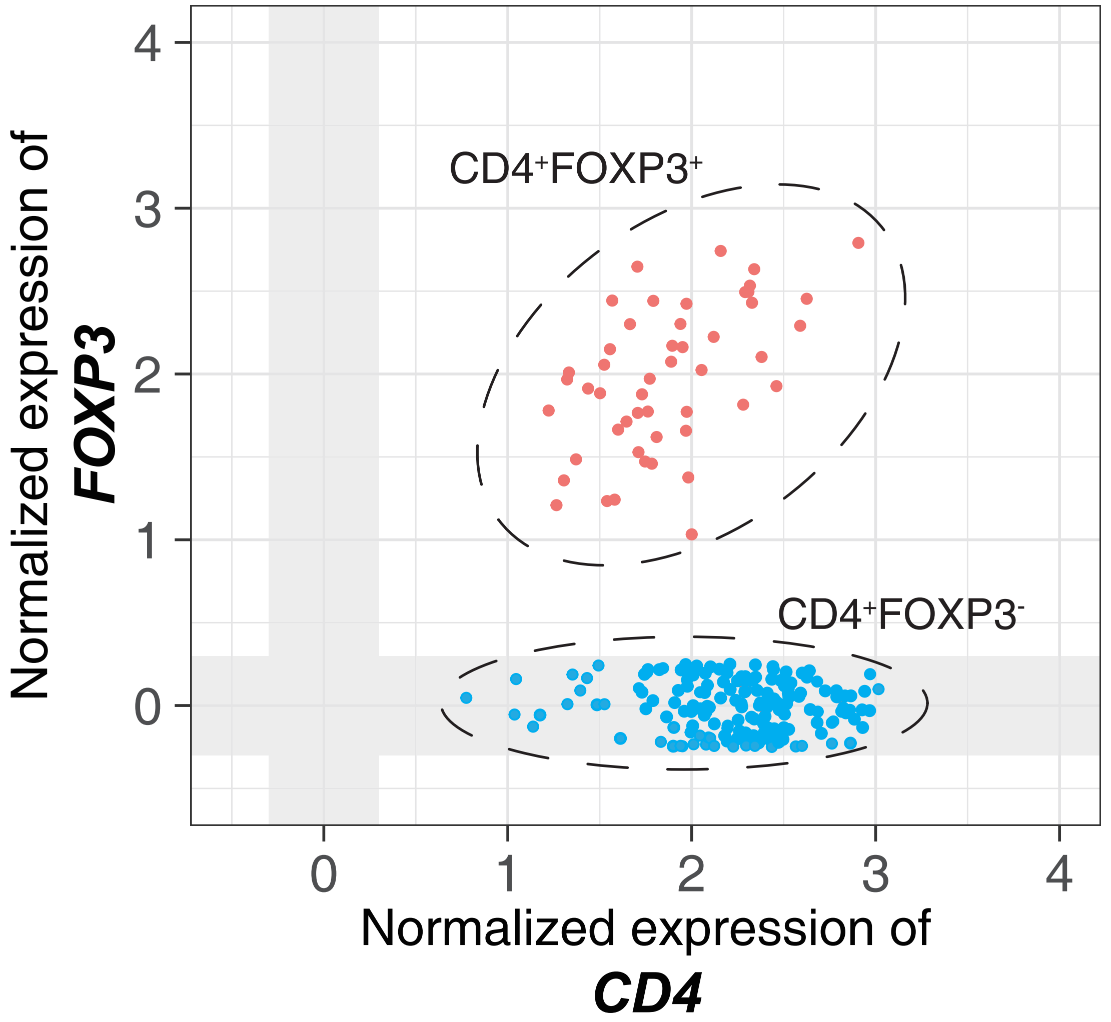

Supplemental Figure S6. Dot plots show gene expression of *CD4* and *FOXP3* for the CD4+ T cells of the subcutaneous lesion sample of the SPTCL patient studied. CD4+FOXP3+ T cells were colored in red, CD4+-FOXP3- T cells were colored in blue. We calculated the percentage (21.97%) of CD4+FOXP3+ T cells in CD4+ T cells to estimate the proportion of Tregs in subcutaneous lesion.

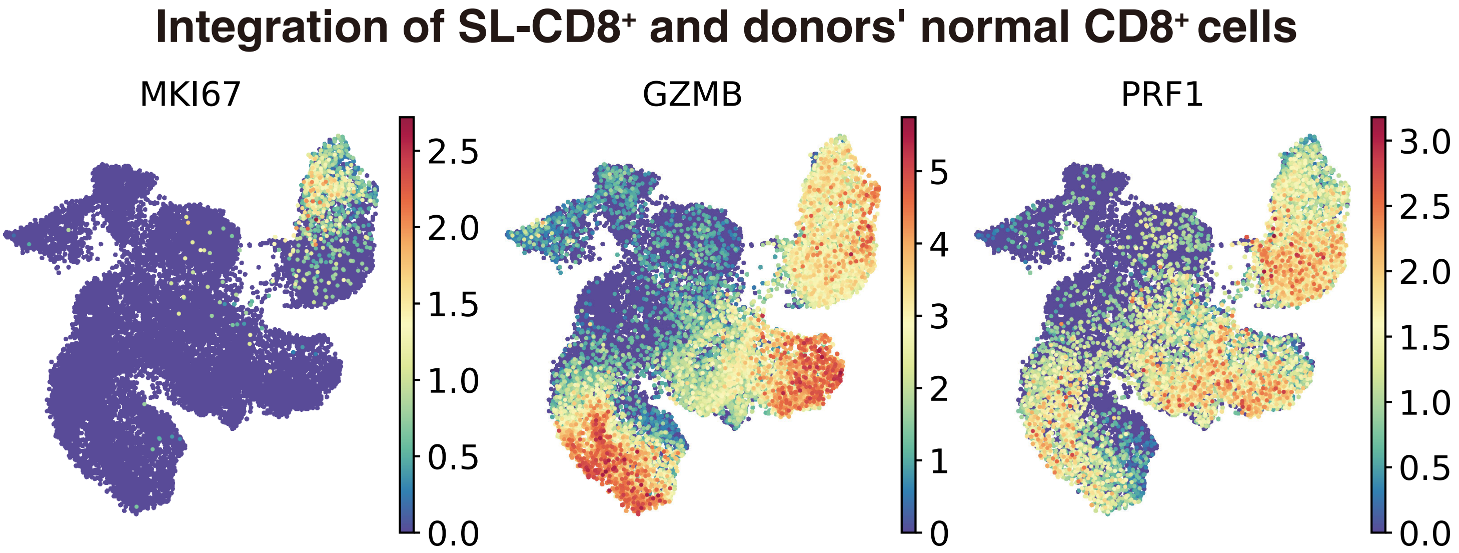

Supplemental Figure S7. Integration UMAP projects of known markers used to diagnose SPTCL. SL, subcutaneous lesion.

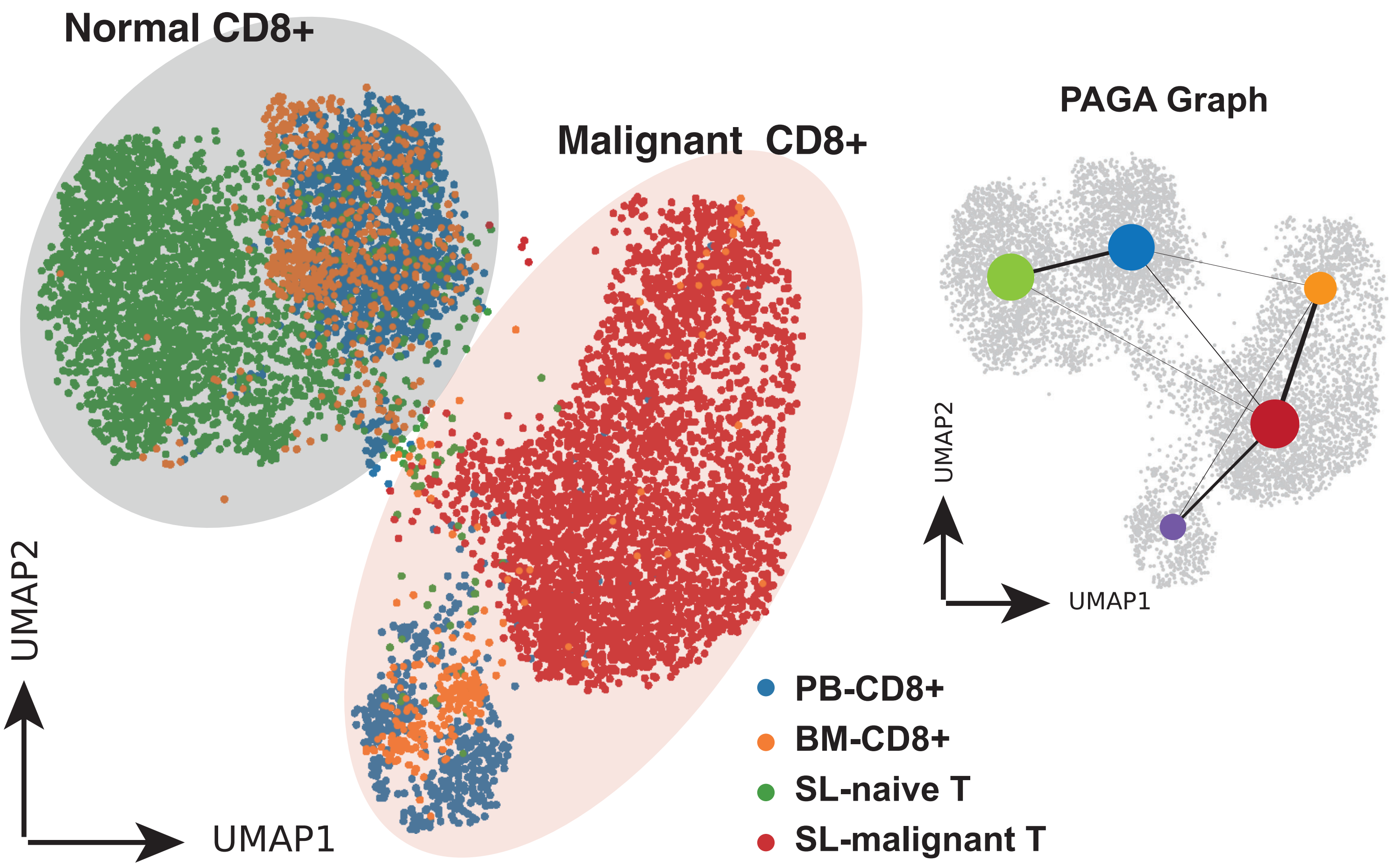

Supplemental Figure S8. Transcriptomic comparison of CD8⁺ T cells from the patient peripheral blood, bone marrow, tumor tissue, related to Figure 3. (A) UMAP projection of patient sample with normal CD8⁺ T cells outlined in grey and malignant CD8⁺ cells in orange. BM, bone marrow; PB, peripheral blood; SL, subcutaneous lesion. **(B)** Cells are colored with various clustering by the Leiden algorithm. Results of partition-based graph abstraction (PAGA).

Integration of SL-CD8$^+$ cells and matched PB/BM CD8$^+$ cells

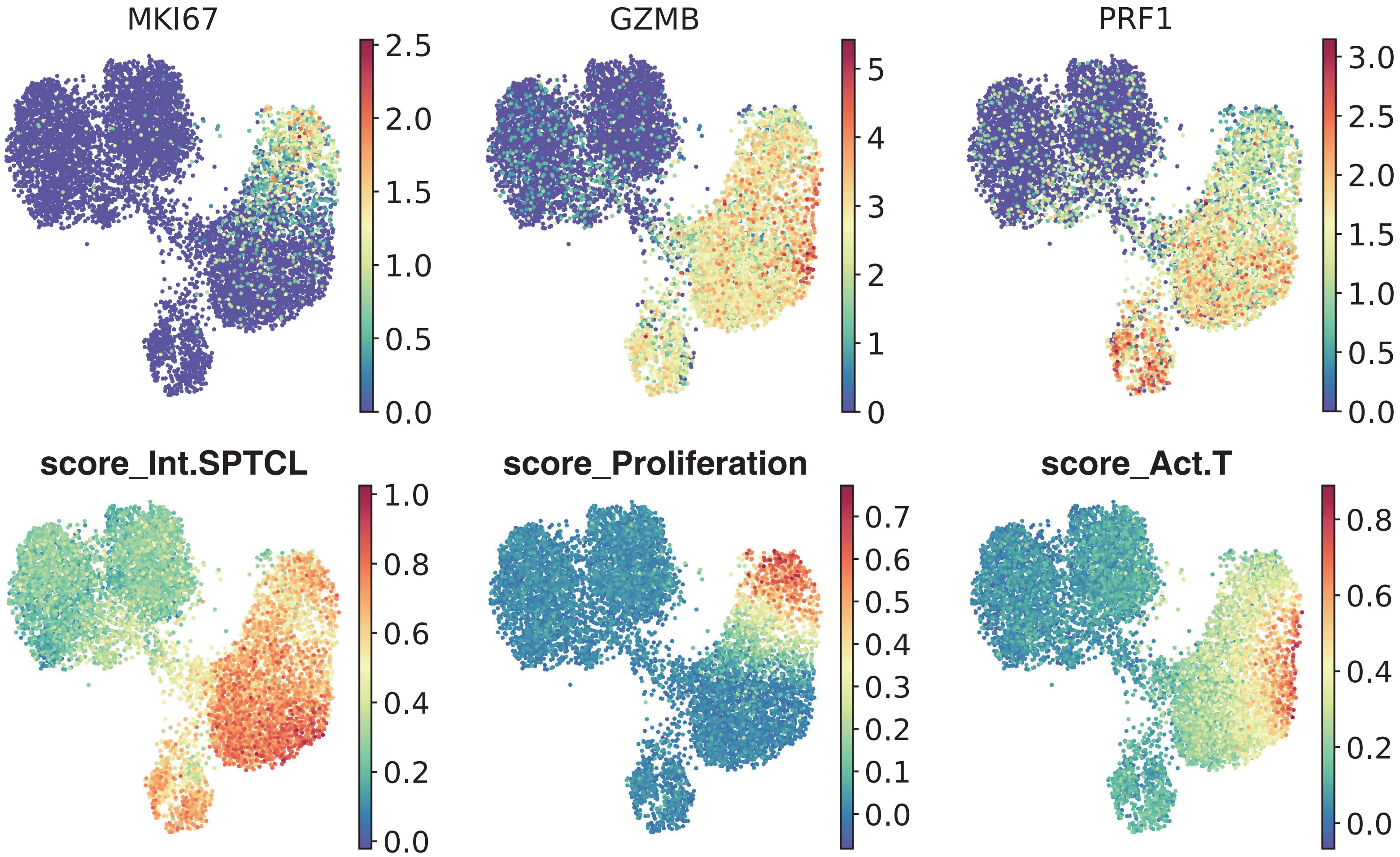

Supplemental Figure S9. Results of known markers and GEP-program cell scores in UMAP space (first two dimensions). BM, bone marrow; PB, peripheral blood; SL, subcutaneous lesion.

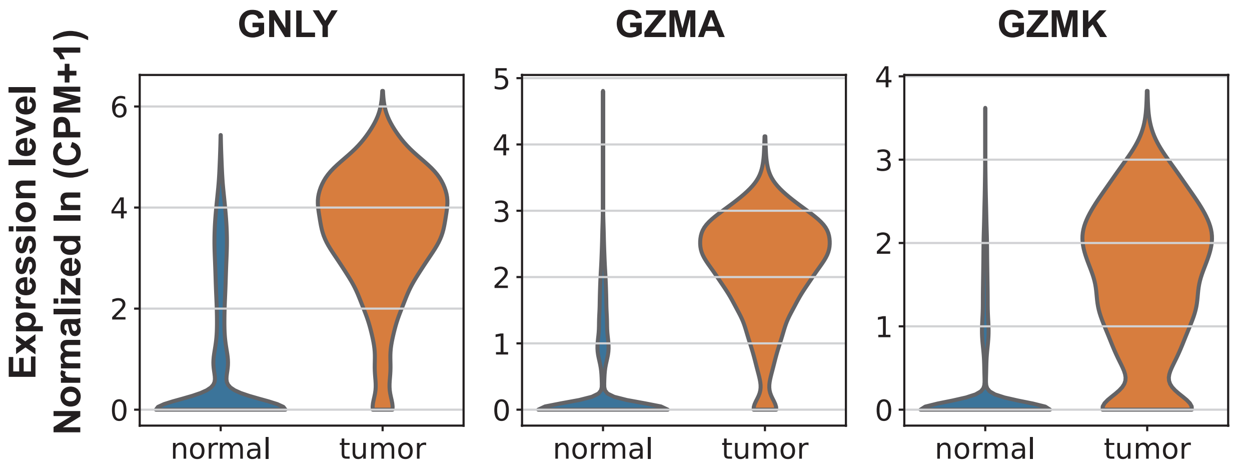

Supplemental Figure S10. Violin plots of known markers of SPTCL (adjusted P < 1e-10).

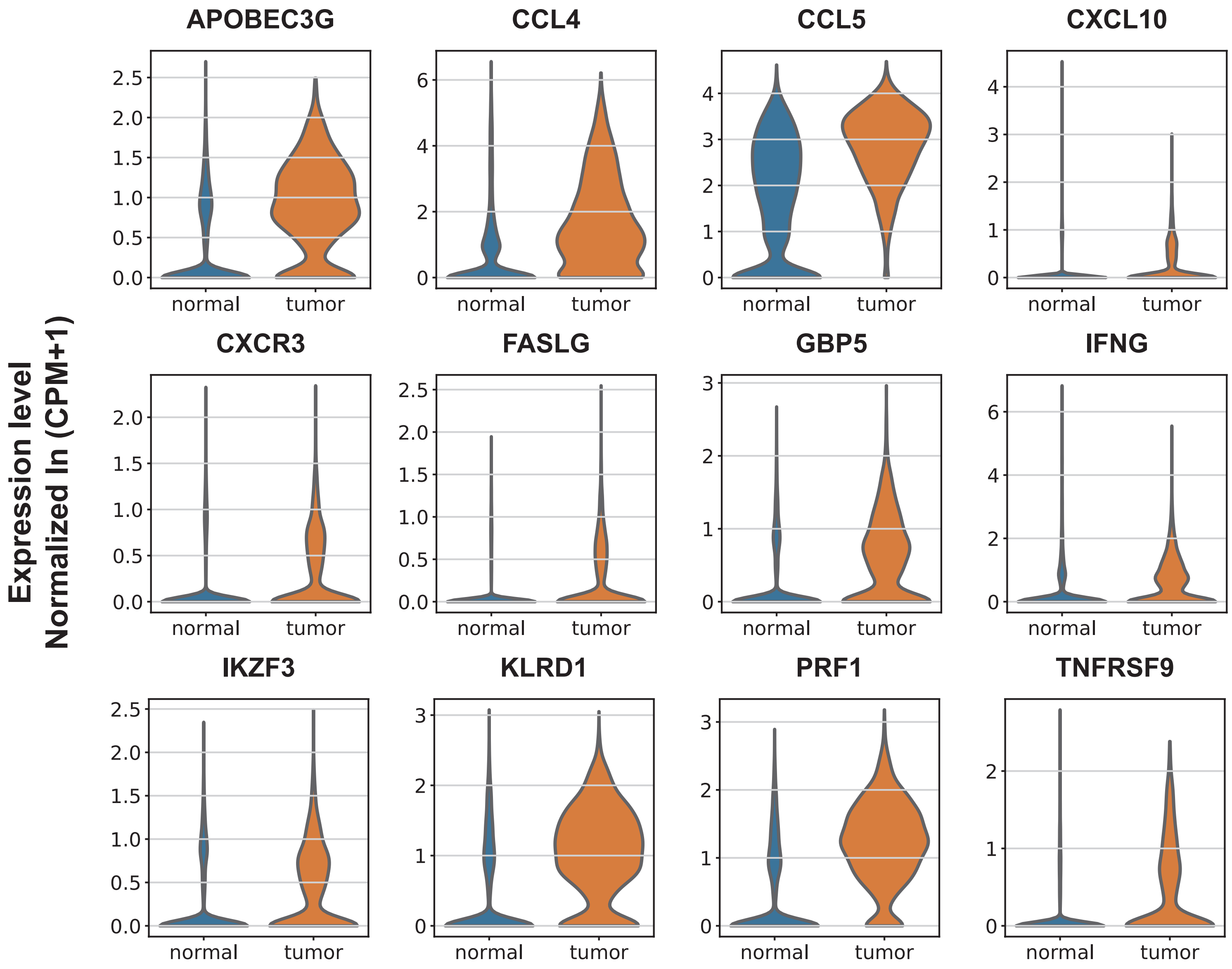

Supplemental Figure S11. Violin plots showing previously identified gene expression of SPTCL cells (adjusted P < 1e-10).

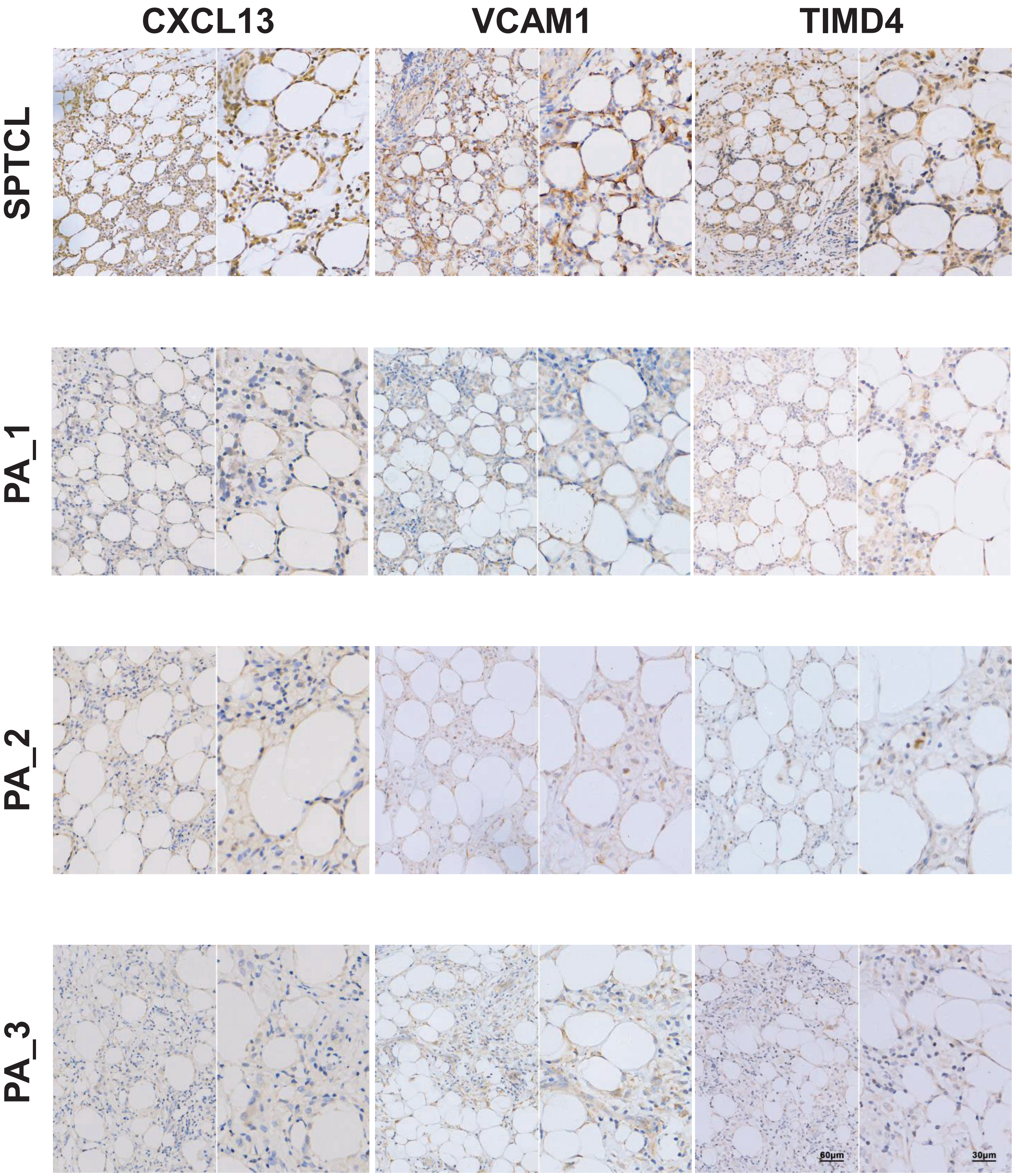

Supplemental Figure S12. Verification results of three novel markers (VCAM1, CXCL13, TIMD4). (A) Immunohistochemical stain from subcutaneous lesion biopsies of Subcutaneous panniculitic-like T-cell lymphoma (SPTCL, n=1), and panniculitis (PA, n=3), each at 20x (left) and 40x (right).
